# Parental Effect of Long Acclimatization on Thermal Tolerance of Juvenile Sea Cucumber *Apostichopus japonicus*

**DOI:** 10.1371/journal.pone.0143372

**Published:** 2015-11-18

**Authors:** Qing-lin Wang, Shan-shan Yu, Yun-wei Dong

**Affiliations:** 1 State Key Laboratory of Marine Environmental Science, College of Marine and Earth Sciences, Xiamen University, Xiamen, P.R. China; 2 Beidaihe Central Experiment Station, Chinese Academy of Fishery Sciences, Qinhuangdao, P.R. China; University of Connecticut, UNITED STATES

## Abstract

To evaluate the thermal resistance of marine invertebrates to elevated temperatures under scenarios of future climate change, it is crucial to understand parental effect of long acclimatization on thermal tolerance of offspring. To test whether there is parental effect of long acclimatization, adult sea cucumbers (*Apostichopus japonicus*) from the same broodstock were transplanted southward and acclimatized at high temperature in field mesocosms. Four groups of juvenile sea cucumbers whose parents experienced different durations of high temperature acclimatization were established. Upper thermal limits, oxygen consumption and levels of heat shock protein mRNA of juveniles was determined to compare thermal tolerance of individuals from different groups. Juvenile sea cucumbers whose parents experienced high temperature could acquire high thermal resistance. With the increase of parental exposure duration to high temperature, offspring became less sensitive to high temperature, as indicated by higher upper thermal limits (LT_50_), less seasonal variations of oxygen consumption, and stable oxygen consumption rates between chronic and acute thermal stress. The relatively high levels of constitutive expression of heat-shock proteins should contribute to the high thermal tolerance. Together, these results indicated that the existence of a parental effect of long acclimatization would increase thermal tolerance of juveniles and change the thermal sensitivity of sea cucumber to future climate change.

## Introduction

Environmental alterations related to global change have significant impacts on biodiversity and thus on many aspects of community and ecosystem functioning [1–3]. The resilience and resistance of ecosystem function to perturbations are closely related to organisms’ phenotypic plasticity in both temporal and spatial scales [4–6]. Besides fixed effects (local adaptation), local acclimatization contributes importantly to heat tolerance, as recently shown for reef coral resistance to future increases in sea temperature [7]. Therefore, it is crucial to investigate the impacts of acclimatization on the resilience and resistance to changing environments.

Changes in temperature on seasonal or even diurnal time-scales lead ectothermic animals to make acclimatory responses, which include the detection of environmental signals, the transduction of these signals into a cellular response, and the activation of the molecules that cause a change in phenotype [8,9]. Stress responses play an important role in shaping species distributions and robustness to climate change [10]. Moreover, many aquatic organisms have developed capacities for thermal acclimatization that provide greater tolerance to exposure to stressful temperatures [11,12]. Although acclimatization usually occurs within an individual’s lifetime, its effects may sometimes persist for several generations [13]. Therefore, acclimatization responses of individuals can provide fitness advantages in a population over generations. The potential mechanistic bases of this phenomenon include parental supply to offspring of nutrients, hormones, mRNA, or other factors that alter the physiological state at birth [14]. This situation has been well studied in bacteria and insects [13,15–17]. However, few studies focus on sea cucumber *Apostichopus japonicas*, one of the most important aquaculture species in China.

Sea cucumber aquaculture is a thriving and prosperous industry, with a production value of $3.2 billion USD in 2013 [18]. This species has an average lifespan of 8–10 years, and the age for sexual maturity is 2–3 years. It is naturally distributed in northern China, and the southmost distribution lies in Ping Island (35°05'N, 119°53'E), Shandong Province. The thermal tolerance range of *A*. *japonicus* is from ~0°C to ~30°C, and the optimum temperature for growth is 15–23°C [19,20]. Previous studies show that *A*. *japonicus* is sensitive to high temperature, and adults enter aestivation when water temperature is about 20~24.5°C [21,22]. This aestivation usually lasts about three months in northern China and most individuals are in a state of fasting, inactivity and low metabolism [21]. During summer, large scale mortality occurs and, after aestivation, surviving sea cucumbers lose more than half of their body weight in autumn. Acute heat shock can improve the thermal tolerance of *A*. *japonicus*. After 2-h heat shock at sublethal temperature (30°C), juvenile sea cucumbers could acquire higher thermal tolerance than those that didn’t experience sublethal heat shock, indicating the existence of plasticity of thermal tolerance in this species [23].

Thermal acclimatization can affect numerous physiological traits that are closely related to ecological fitness. Previous studies have shown that critical temperature and metabolism are strongly influenced by the thermal history [24–27]. Oxygen consumption is an important physiological trait and is proposed to be the determining factor for thermal tolerance for many ectotherms [28]. The induction and up-regulation of heat shock proteins (Hsps) occurs as a cellular defense against thermal stress, and they are crucial for maintaining cellular protein stability and resistance to heat stress [29]. The expression levels and patterns are closely related to animals’ thermal tolerance, distribution and population dynamics [30–33].

To understand the mechanism of the resistance of marine invertebrate to elevated temperatures under scenarios of future climate change, it is important to know the parental effect of long acclimatization on thermal tolerance of juveniles. If offspring whose parents experience high temperature acclimatization can acquire higher thermal tolerance, then the negative impact of climate change on population dynamics and ecosystem functioning will be reduced. In the present study, we hypothesized that acclimatization of adult sea cucumbers *A*. *japonicus* could change the thermal limits of juveniles, and the modification of thermal tolerance was closely related to the changes in oxygen consumption rates and expression of *hsp*s.

## Methods

### Ethics statement

Both sites at Qingdao and Xiapu were private and the owners gave permission to conduct the transplantation and the following study. As *A*. *japonicus* is not a protected species, and collections were only made from private enterprises, no other specific permits were required to collect this species from these locations/activities. All procedures involving the collection and sampling of sea cucumber during this study were approved by the Animal Care and Use committee of State Key Laboratory of Marine Environmental Science at Xiamen University.

### Collection and acclimatization of animals

**Fig 1:**
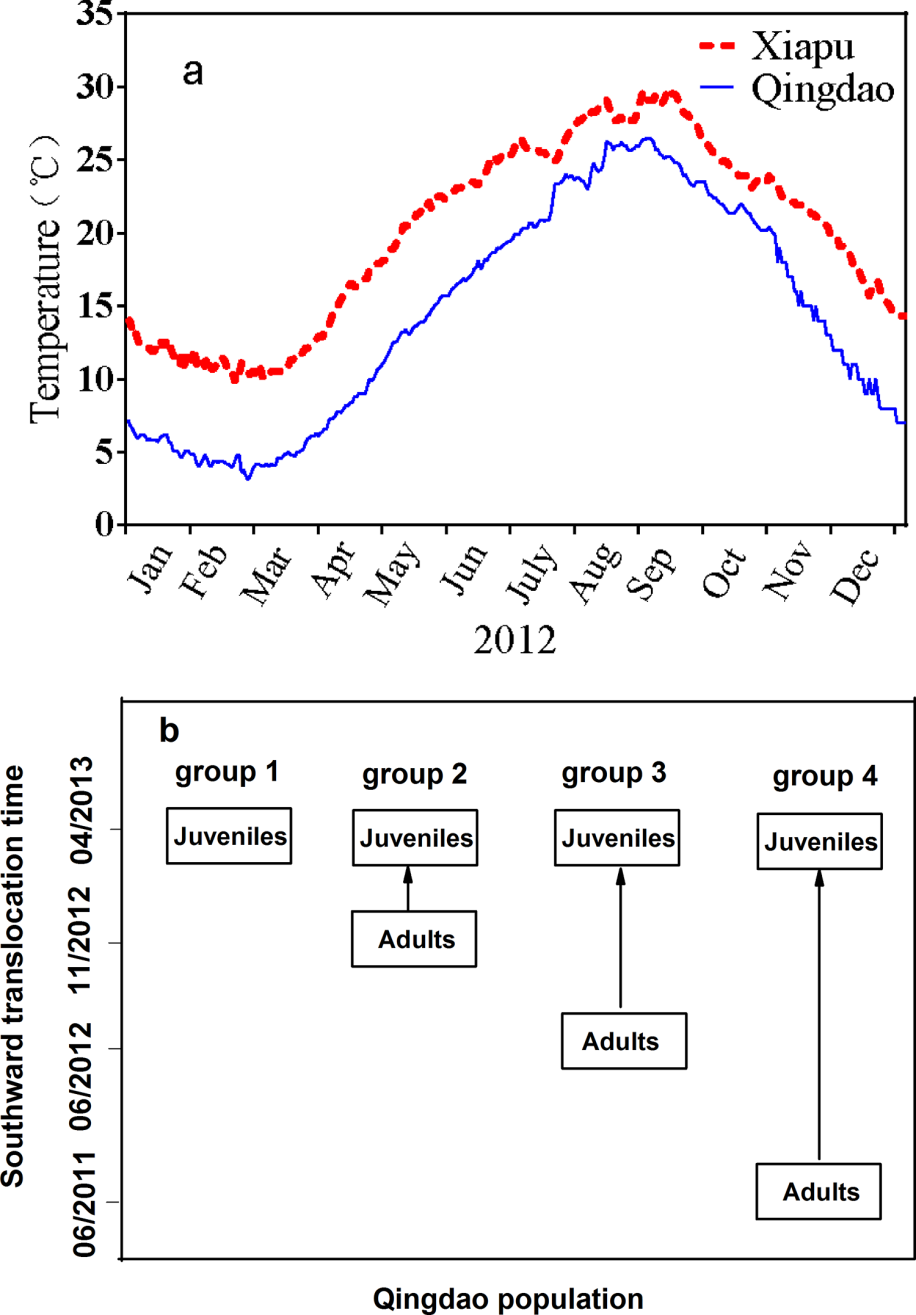
(a) Water temperature in Xiapu and Qingdao. Water temperatures were measured daily from January to February 2012; **(b) a scheme representing the thermal history of the different groups** (see text for details).

Adult sea cucumbers (mean body weight > 200 g) from the same broodstock were collected from an aquaculture facility in Qingdao (36°21'N, 120°43'E) and transplanted to Xiapu (26°41'N, 120°06'E) using cool boxes to keep the temperature below 20°C (Fig 1A). Transplanted animals were reared in the traditional multi-tier baskets (S1A Fig), each basket consisting of six vertically connected tiers (S1B Fig), the inside surface of a tier being 0.486 m^2^. Then they were hung on fish raft in shallow water (1–2 m in depth). Two animals with total wet weight of ~ 400 g (the rearing density typically used in the traditional culture system) were stocked per tier, and this rearing density was maintained during the whole period of adult sea cucumber acclimation. For each group, there were more than 10 traditional multi-tier baskets, and 5 of them were randomly chosen to test the mortality of adult sea cucumbers during acclimatization. Sea cucumbers were fed *ad libitum* twice a week with a mixture of seaweed and mashed forage fishes in winter and early spring. When ambient water temperature was beyond 20°C, sea cucumbers entered a state of aestivation. At that time, there was no need for feeding.

### Groups with different thermal histories

According to the thermal history of adult sea cucumbers, four different groups were constructed (Fig 1B). Animals of group 1 were juveniles that were transplanted from Qingdao to Xiapu in April 2013; individuals of group 2, group 3 and group 4 were descendants of the adults that were transplanted from Qingdao to Xiapu in November 2012, June 2012 and June 2011, respectively. The duration of acclimatization of adults was gradually extended from group 1 to group 4, and group 4 was the longest-acclimatization group.

### Artificial breeding and larvae rearing

The artificial breeding of group 2, group 3 and group 4 was carried out in March 2013 in Xiapu. For each group, in total 50 well-developed adult sea cucumbers (mean wet weight > 400 g) were chosen and induced to spawn applying the following procedures. The holding tank (6.0 m × 1.0 m × 1.5 m, length × width × height) was drained completely leaving the sea cucumbers to dry for 40 min, and then the individuals were sprayed 10 min with 18°C seawater. Then the holding tank was filled with fresh 21°C seawater. Usually, the broodstock began to spawn 2 h after spraying.

After spawning, the broodstock were removed, while fertilization took place in the water. The eggs were washed several times to remove excessive sperms [34], and the fertilized eggs’ density was adjusted to 10–20 individuals ml^-1^. After the hatching of oosperms, larvae were transferred to outdoor mesocosms (6.0 m × 2.0 m × 1.5 m, length × width × height). For each group, there were 5 mesocosms, and the rearing density was maintained at 0.5 individual ml^-1^ [35]. From the pre-auricularia stage, larvae were fed on a mixture of *Chaetoceros muelleri*, *Dunaliella salina* and marine red yeast. From pre-auricularia to post-auricularia stage, algal cells in the rearing water gradually increased from 1.0 × 10^4^ cells ml^-1^ to 2.5 × 10^4^ cells ml^-1^. Feeding rates were based on feeding activity of the larvae and concentrations of the algae in water. During the whole culture period, larvae were reared in natural seawater, and aeration was provided continuously. Natural seawater was filtered through a sand filter (salinity 30–32 ppt), and one-half to two-thirds of the rearing water was replaced daily. Water pH was about 7.8 and ammonia was less than 0.24 mg L^−1^. Water temperature, salinity, pH and ammonia were determined with a mercury thermometer (accuracy ± 0.2°C), salinity refractometer (AIAGO, Japan), pH meter (PH3150i, WTW, Germany), and hypobromite oxidation methods [36], respectively.

In each group, there were > 20 million of juveniles. All juveniles of the four groups were randomly distributed into 20 outdoor mesocosms, and each group had 5 replicates. Mortalities of juvenile sea cucumbers were assessed during rearing. After at least a 2-month culture period in Xiapu, juveniles in each group were randomly collected for further physiological measurements.

### Lethal temperatures in different groups

#### The temperatures at which 50% of the sea cucumbers died (LT_50_)

Upper thermal limits in all the groups were assessed using a method previously described [22]. A total of 720 individuals were used in these studies (4 groups × 6 temperatures × 3 replicates × 10 individuals). The initial body weight of these animals was 0.32 ± 0.15 g (Mean ± SD), and individuals of essentially identical weight were assigned at random to 72 aquaria (10 individuals / aquarium). Ten specimens in each group (body weight: 0.32 ± 0.15 g, Mean ± SD) were used at each of the six temperatures (29, 30, 31, 32, 33 and 34°C). Three replicates were applied for each group and each temperature. Individuals were placed into a 2-l glass beaker. Water temperature in the beaker was adjusted to the designated values using a water bath (HBS-1000, EYELA, Tokyo, Japan). The water temperature was recorded every 5 min. After 2 h of heat shock, sea cucumbers were returned to the natural seawater (~23°C) for 7 days, and mortalities were recorded. The temperatures at which 50% of the sea cucumbers died (LT_50_) and the 95% confidence limits were calculated using Probit analysis using SPSS.

#### Induced thermal tolerance in group 1 and group 2

Because there was no significant difference in LT_50_ between group 1 and group 2, an experiment was designed to measure whether there was significant difference in induced thermal tolerance between them. Based on the results from LT_50_, 29°C and 32°C were selected as the sublethal and lethal temperature, respectively. The temperature control method was similar to that in the experiment on thermal tolerance limits. A total of 60 individuals (body weight: 0.33 ± 0.14 g, Mean ± SD) from each group were exposed to sublethal temperature (29°C). After 2 h of heat shock, they were returned to natural water (~23°C) for 24 h recovery. Then, 30 individuals (3 replicates × 10 individuals) were divided into three subsets and exposed to lethal temperature (32°C) for 2 h of heat shock. Subsequently, these animals were returned back to natural water for 7 days to assess mortalities. Thirty sea cucumbers (3 replicates × 10 individuals) from each group that experienced sublethal heat shock (NO-LHS) were transferred into natural seawater for 7 days to assess the mortality of juvenile *A*. *japonicus* which only encountered heat shock of 29°C.

Thirty individuals (3 replicates × 10 individuals) of each group without previous sublethal heat shock (NO-SHS) were exposed to lethal heat shock (LHS: 32°C, 2 h) and returned to natural seawater for 7 days to assess survival rate.

### Oxygen consumption

#### Seasonal difference of oxygen consumption rates (OCR) among groups

Ambient water temperature in Xiapu increased to ~23°C, ~26°C and ~29°C in June, July and August, respectively. In June, July and August, OCRs were measured in different groups. Prior to the test of oxygen consumption, juveniles were starved for 24 h to reduce associated metabolic responses to dietary state. The tested animal was put into a 330 ml conical flask with a rubber plug individually, which was immersed into a water bath (HBS-1000, EYELA, Tokyo, Japan) for temperature control. There were three replicates. Two blank controls to correct for the effect of the respiration of bacteria in the water were used. Oxygen content of water samples was determined using the dissolved oxygen analyzer (YSI 5000; YSI, Yellow Spring, OH, USA), and the OCR of the sea cucumber was calculated from,

$$OCR\;(\mu g\;O_{2}\cdot h^{-1}\cdot g^{-1})\;=\;(D_{t}\;V_{t}-D_{0}\;V_{0})/W\;T$$ (1)

where D_t_ represents the changes of the oxygen content (μg O_2_·L^−1^) before and after test in the test bottles; D_0_ is the changes of the oxygen content (μg O_2_·L^−1^) before and after test in the blank bottles; V_t_ and V_0_ are volumes of the test bottles and blank bottles (L); W is the wet weight of the sea cucumber (g); T is time duration (h).

#### Difference of oxygen consumption rates (OCR) among different groups after acute heat shock

Temperatures were increased at a rate of 3°C h^-1^ from 23°C to 29°C, and then maintained at 29°C for 2 h using a water bath (HBS-1000, EYELA, Tokyo, Japan). After that, OCRs of the four groups (each group had 3 replicates) were measured using the methods described above.

### Gene expression of *hsp*70 and *hsp*90

When the ambient water temperature increased to 23°C, an experiment was carried out to compare expression of genes encoding heat shock proteins (*hsp*s) among the four groups. A total of 30 juveniles (body weight: 0.13 ± 0.04 g, Mean ± SD, 3 replicates × 10 individuals) in each group were randomly distributed into three 1L beakers and water temperature was increased at a rate of 3°C h^-1^ from 23°C to 29°C using a water bath. After exposure at 29°C for 2 h., specimens were immediately frozen in liquid nitrogen for analyses of gene expression. Juveniles without heat stress were also sampled when ambient water temperature increased to 23°C, 26°C and 29°C in June, July and August, respectively.

Total RNA was isolated from ~50 mg of body wall using Trizol Reagent (Invitrogen, Carlsbad, CA, USA). The first strand of cDNA was synthesized using total RNA as a template. Reverse transcriptase (RT) reactions were performed using a PrimeScript™ RT reagent Kit with gDNA Eraser (TAKARA, Shiga, Japan).

In order to compare the relative levels of expressions of *hsp*s between differing groups, three housekeeping genes (β-actin, 18S rRNA and Cytb) and two target genes (*hsp*70 and *hsp*90) were amplified with the same cDNA samples using primers as shown in S1 Table. PCR was carried out in a Bio-Rad CFX96 Real-Time PCR system (Bio-Rad, Hercules, CA, USA) in a 20 μl reaction volume containing 10 μl of 2 × FastStart DNA Universal SYBR Green Master (Roche, Grenzach-Wyhlen, Germany), 1 μl of each primer (10 nmol per μl), 1 μl of cDNA template and 7 μl of RNase-free water. The PCR program was 95°C for 30 s, followed by 40 cycles of 95°C for 10 s, 60°C for 25 s and 72°C for 25 s. All samples were measured in triplicate. Ct (dR) values were analyzed using the Bio-Rad CFX96 System Software (Bio-Rad, Hercules, CA, USA). The expression of *hsp*70 and *hsp*90 mRNA for different treatments was determined relative to the mean value of β-actin, 18S rRNA and Cytb.

### Statistical analysis

The data were analyzed using SPSS for Windows (Version 13.0; SPSS, Chicago, IL, USA). Data were tested for homogeneity of variances using the Levene’s Test. One-way ANOVA followed by post hoc Tukey multiple range test was applied to analyze the differences in mortality during acclimatization period among different groups. The *hsp*70 and *hsp*90 data were log transformed to satisfy the requirement of homogeneity of variances. The differences in OCR, *hsp*70 and *hsp*90 in different seasons (June, July and August) and groups were analyzed using Two-way ANOVA followed by post hoc Tukey multiple range test. One-way ANOVA followed by post hoc Tukey multiple range test was applied to analyze the differences in mortality during acclimatization period, survival rate, OCR, *hsp*70 and *hsp*90 among different groups after acute heat shock. Difference in induced thermal tolerance between group 1 and group 2 was analyzed using independent samples t-test, respectively. Differences were considered significant at *P* < 0.05.

## Results

### Mortality during acclimatization

Mortalities of adult and juvenile sea cucumbers were assessed during the acclimatization period (S2 Table, S3 Table). One-way ANOVA results showed that there were significant differences in mortality of adults among group 2 (15.002 ± 9.129%), group 3 (31.666 ± 9.128%) and group 4 (41.666 ± 8.335%) during acclimatization (*F*_2, 14_ = 11.526, *P* = 0.002). But no significant difference was found over the period in which groups 3 and 4 were acclimating together with group 2 (*F*_2, 14_ = 0.153, *P* = 0.860). During the two-month culture of juveniles, no significant difference in mortality was found among the four groups (*F*_3, 19_ = 0.196, *P* = 0.898).

### Lethal temperatures

The survival rate of juvenile sea cucumbers decreased as exposure temperature increased from 29°C to 34°C (S4 Table). Probit analysis showed that LT_50_ values (95% confidence limits) were 30.659°C (30.443, 30.877°C), 30.661°C (30.454, 30.872°C), 31.146°C (30.939, 31.347°C) and 31.842°C (31.593, 32.089°C) in group 1, group 2, group 3 and group 4, respectively. LT_50_ values of group 3 and group 4 were significantly higher than those of group 1 and group 2. There was no significant difference in LT_50_ between group 1 and group 2, and LT_50_ of group 4 was significant higher than that of group 3 (*F*_3, 11_ = 35.806, *P* < 0.001).

**Fig 2:**
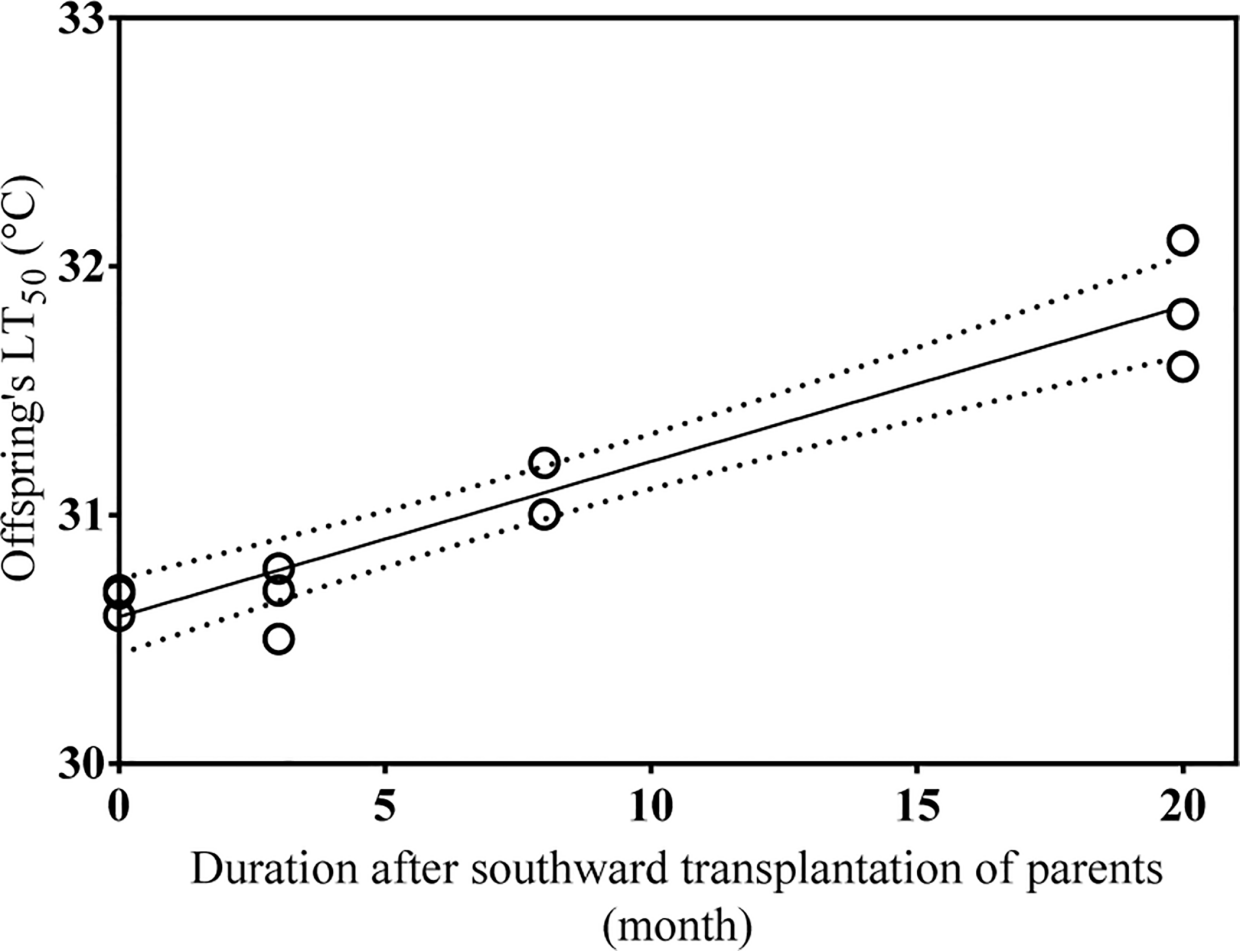
The relationship between offspring LT_50_ and duration (months) after southward transplantation of parents. There was a linear relationship between offspring LT_50_ and duration of transplantation of parents (R^2^ = 0.910, *P* < 0.001). 95% Confidence intervals are shown by dashed lines.

The relationship between LT_50_ values of juveniles and duration (months) after southward transplantation of parents was analyzed. Results showed that there was a linear relationship between them (R^2^ = 0.910, *P* < 0.001) (Fig 2).

**Fig 3:**
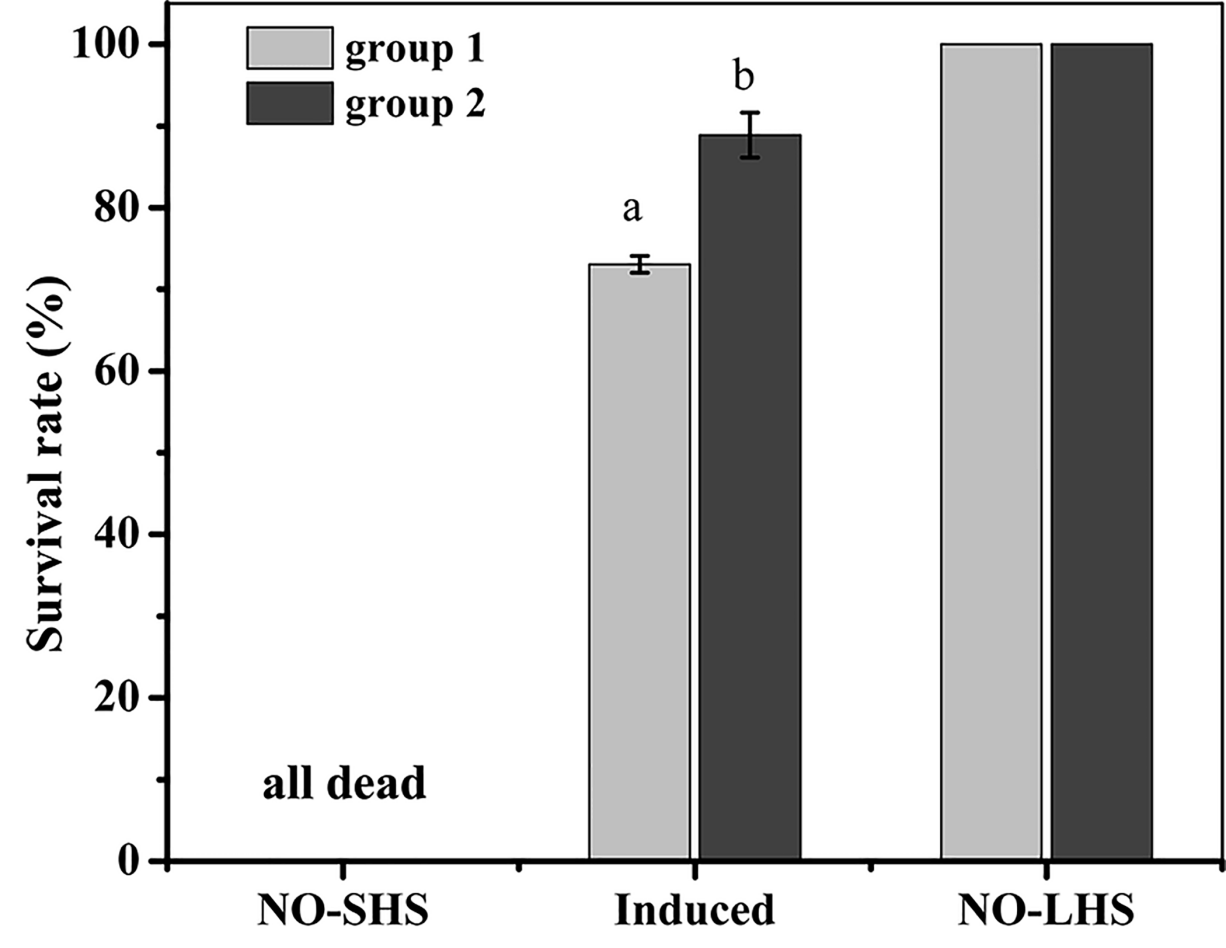
Induced thermal tolerance following exposure to sublethal heat shock (SHS: 29°C) for 2 h. SHS sea cucumbers were incubated at natural sea water for 24 h, and then were given a potentially lethal heat shock (LHS: 32°C 2 h). “NO-SHS” refers to sea cucumbers without sublethal heat shock; “NO-LHS” refers to sea cucumbers given SHS without LHS. Values are mean ± S.E. (n = 3). Means with different letters are significantly different (*P* < 0.05).

After a sublethal heat shock of 29°C, sea cucumbers were returned to natural seawater for 24 h recovery and then were put into 32°C to measure their survival (Fig 3). Juveniles in all groups survived after the exposure at 29°C. Post-recovery survival after a heat shock of 32°C showed the survival rate of group 2 (88.89%) was significant higher than that of group 1 (73.05%, *P* = 0.006).

### Oxygen consumption rates

**Fig 4:**
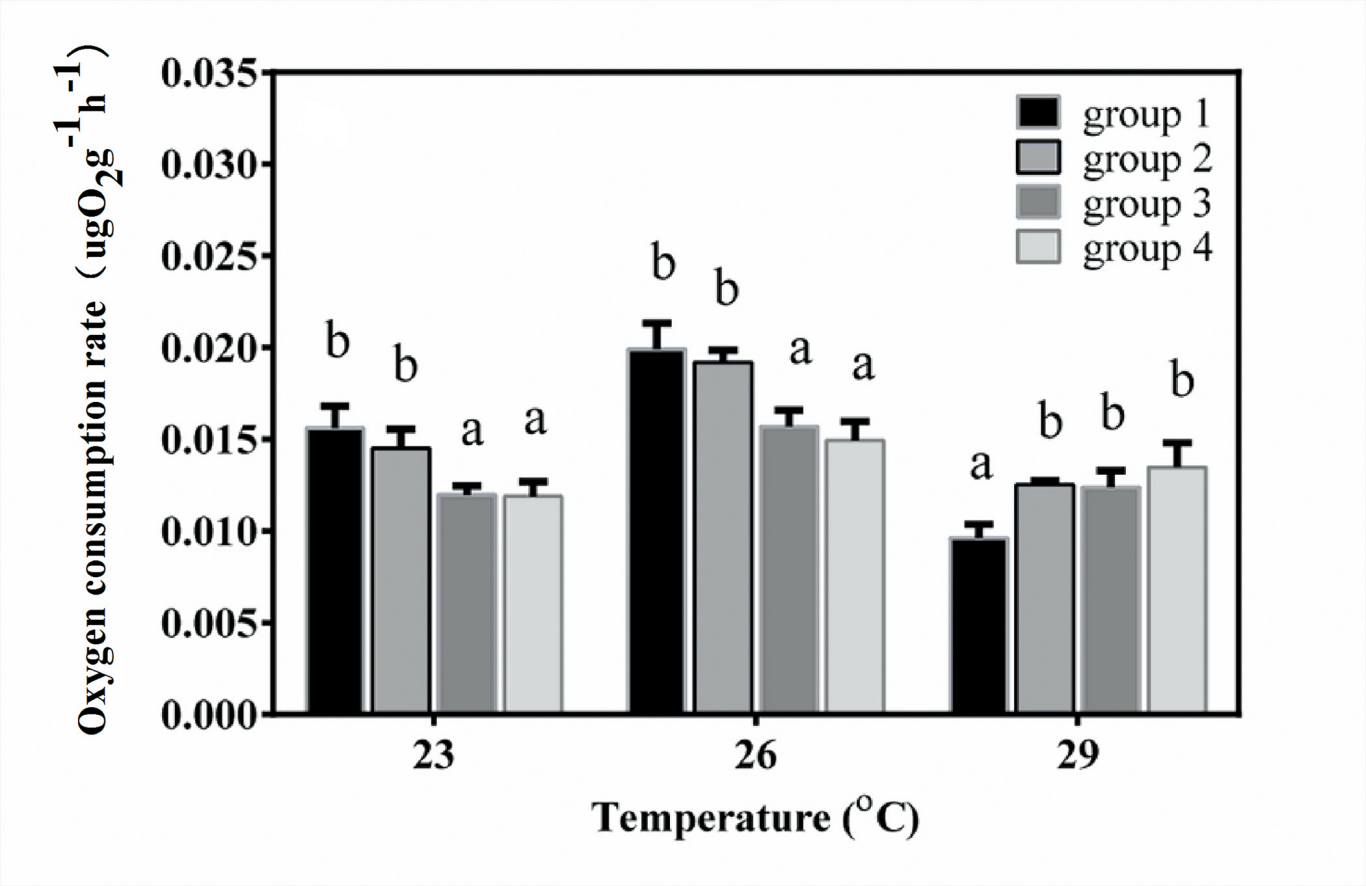
Season variation of oxygen consumption rate of juvenile sea cucumbers. After artificial breeding, juveniles were cultured in outdoor mesocosms. When temperature increased to 23°C in June 2013, 26°C in July 2013 and 29°C in August 2012, oxygen consumption was measured. Values with different letters are significantly different (*P* < 0.05) among different groups at the same temperature.

**Fig 5:**
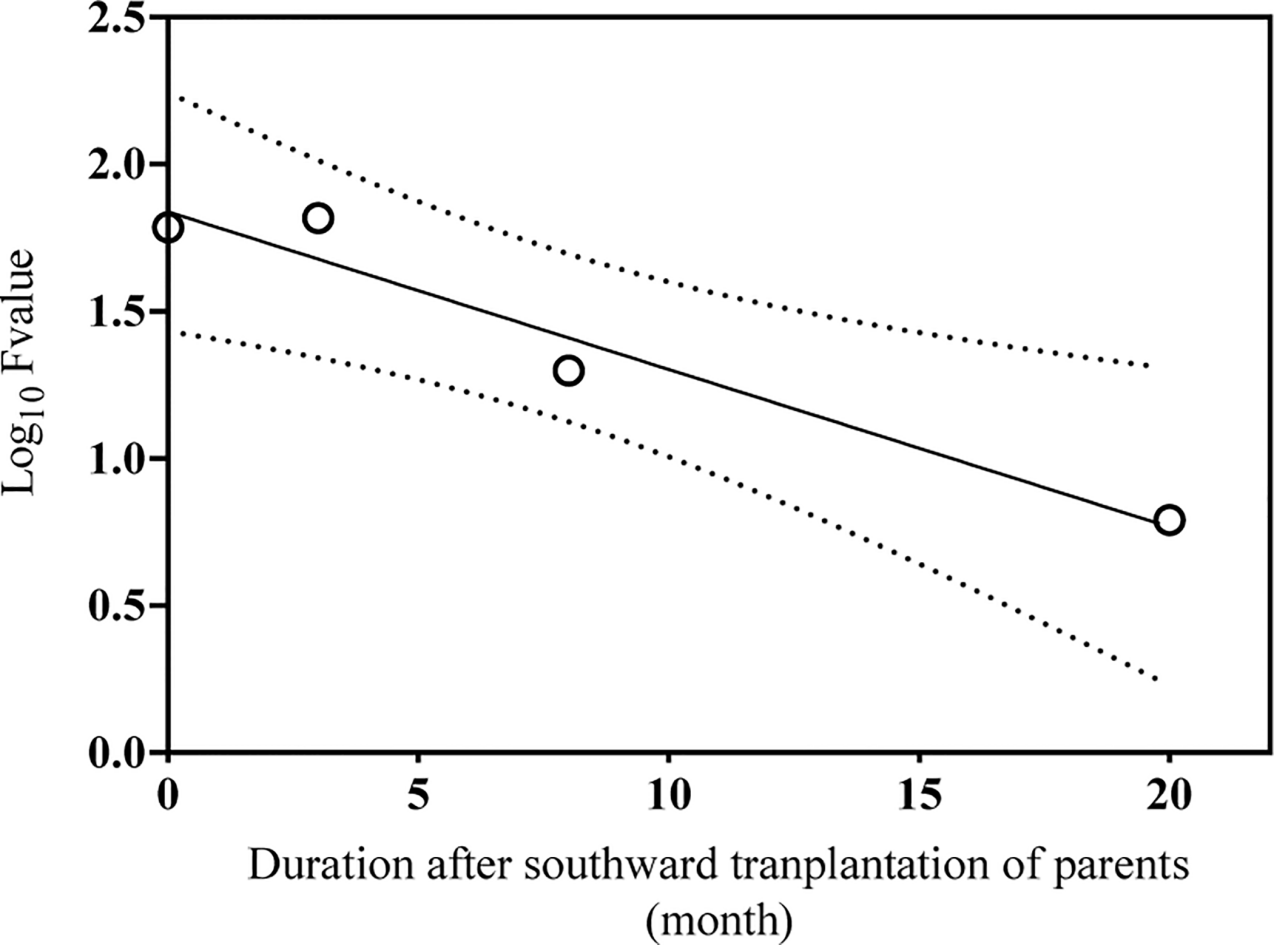
The relationship between offspring seasonal variations of oxygen consumption and duration after southward transplantation of parents. The F values of One-way ANOVA analyses, which were applied to analyze the differences of oxygen consumption among different temperatures within the same group, represent the seasonal variations of oxygen consumption. There was a linear relationship between offspring seasonal variation of oxygen consumption and duration of transplantation of parents (R^2^ = 0.950, *P* = 0.025). 95% Confidence intervals were shown by dashed lines.

The initial and final oxygen concentrations in each measurement are shown in S5 Table. Two-way ANOVA results showed that the oxygen consumption rates (OCRs) were significantly different among the four groups (*F*_3, 36_ = 15.388, *P* < 0.001) and among different temperatures (*F*_2, 36_ = 121.066, *P* < 0.001). There was significant interaction among different temperatures and groups (*F*_6, 36_ = 15.582, *P* < 0.001). At 23°C and 26°C, OCRs of group 1 and group 2 were significantly higher than OCRs of the other two groups; however, OCRs of group 2, group 3 and group 4 were significantly higher than that of group 1 at 29°C (Fig 4). The relationship between offspring seasonal variations of oxygen consumption and duration after southward transplantation of parents was analyzed. Results showed that there was a linear relationship between them (R^2^ = 0.950, *P* = 0.025) (Fig 5).

**Fig 6:**
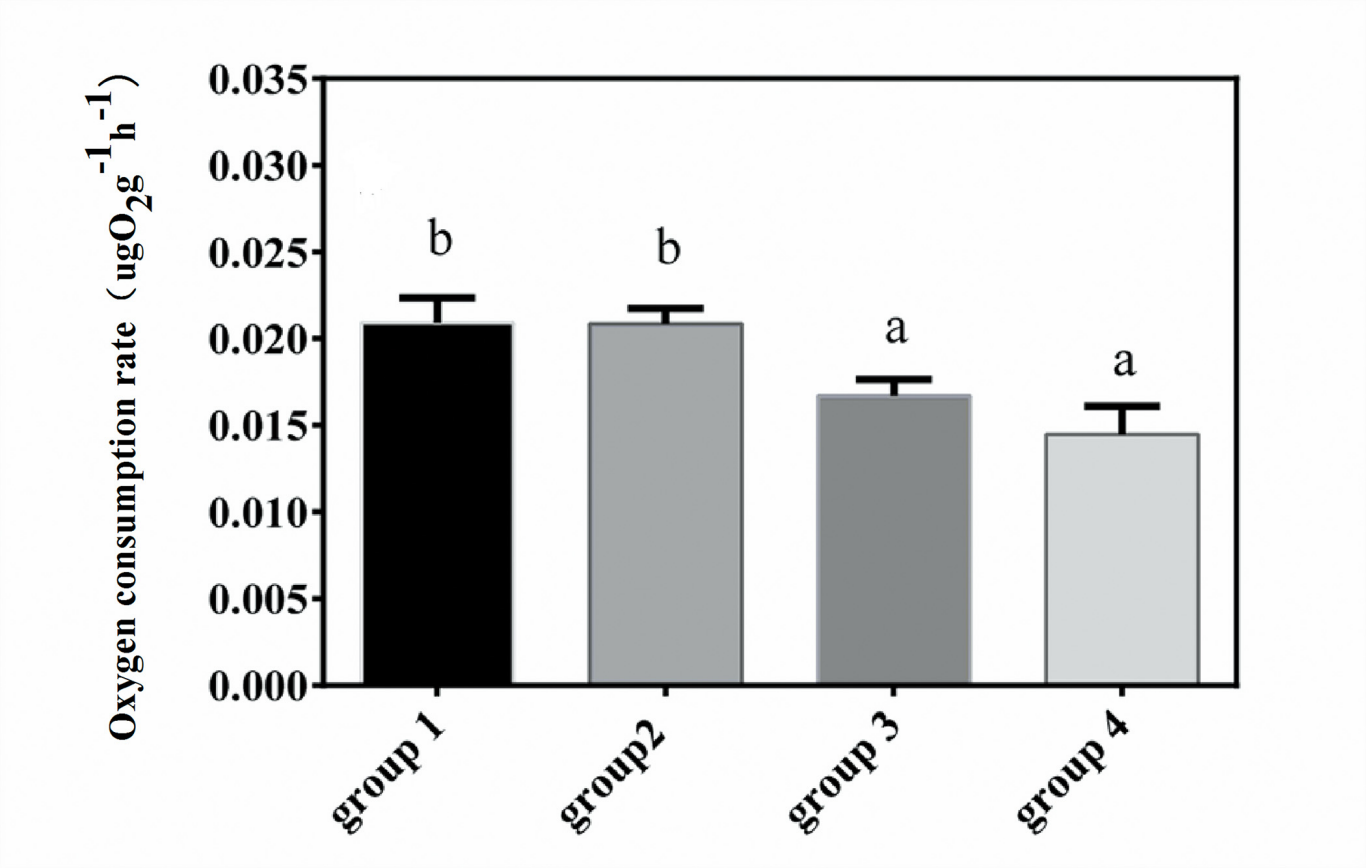
Oxygen consumption rate of juvenile sea cucumbers after acute heat shock. Values with different letters are significantly different (*P* < 0.05). Values are mean ± S.E. (n = 3).

OCRs of the four groups after acute heat shock (transfer from 23°C to 29°C) were significantly different (*F*_3, 11_ = 19.469, *P* < 0.001), and OCRs of group 3 and group 4 were significantly lower than the other two groups (Fig 6). Individuals of group 4 had the lowest OCR (14.481 ± 0.928 μg O_2_ g^-1^ h^-1^).

**Fig 7:**
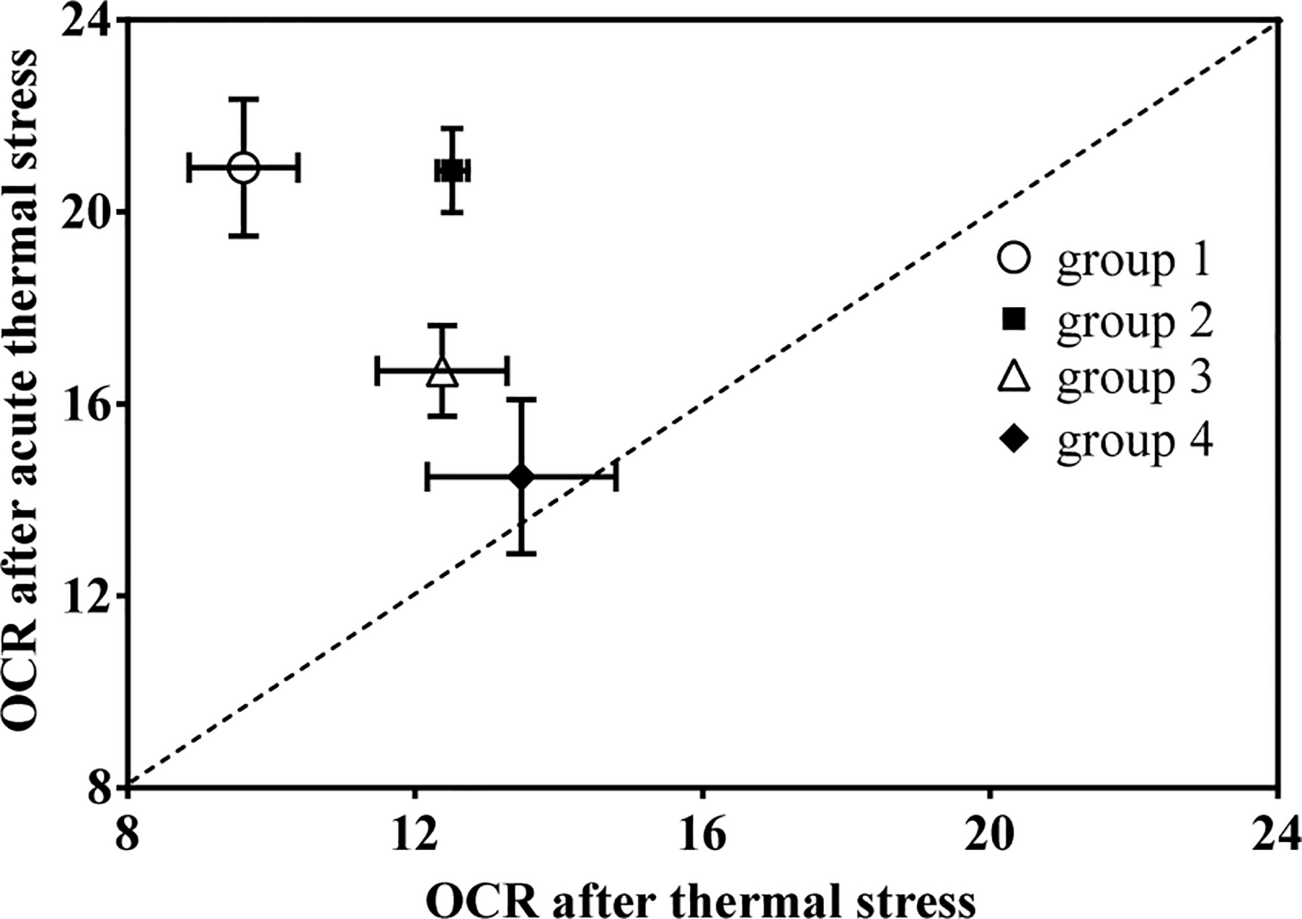
The relationship between oxygen consumption after acute and chronic thermal stress. The temperature increase from 23°C to 29°C between June and August 2012 can be regarded as a chronic thermal stress (OCR_c_); the temperature increase from 23°C to 29°C within 2 h can be regarded as an acute thermal stress (OCR_a_). In group 1 and 2, OCR_a_ was dramatically higher than OCR_c_. However, there was no obvious difference between OCR_a_ and OCR_c_ in group 3 and group 4, especially in group 4.

The relationship between oxygen consumption after acute and chronic thermal stress was analyzed. In the present study, the temperature increase from 23°C to 29°C from June to August 2012 can be regarded as a chronic thermal stress (OCR_c_); the temperature increase used in heat-stress experiments, an increase from 23°C to 29°C within 2 h, can be regarded as an acute thermal stress (OCR_a_). At the same temperature (29°C), the ratios between OCR_a_ and OCR_c_ were different among different groups (Fig 7). OCR_a_s of group 1, group 2 and group 3 were significantly higher than OCR_c_s in the corresponding group (independent t-test: group 1, *P* = 0.001; group 2, *P* = 0.002; group 3, *P* = 0.005). However, there was no significant difference between OCR_a_ and OCR_c_ in group 4 (independent t-test: group 4, *P* = 0.453).

### Heat shock response

**Fig 8:**
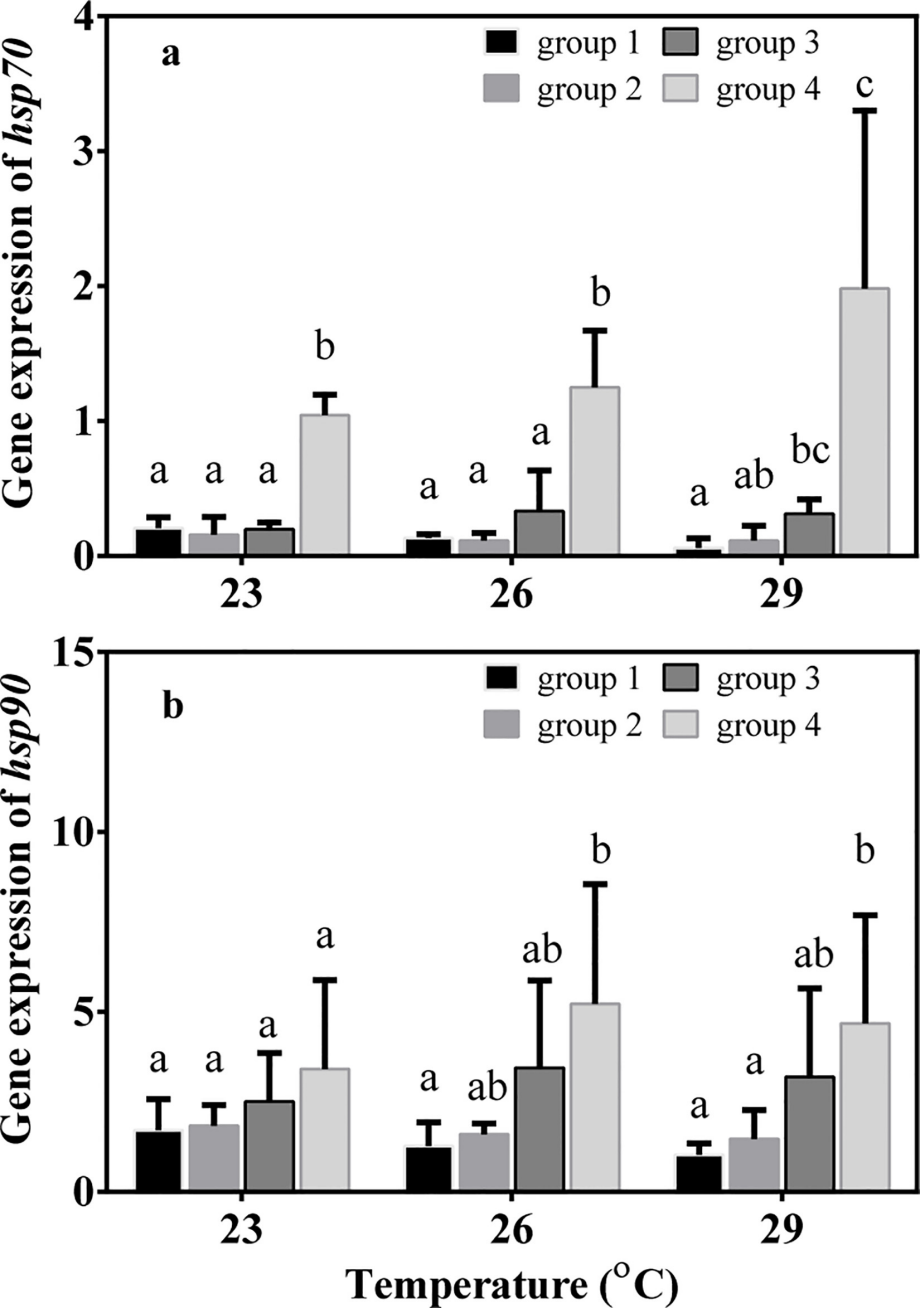
Levels of (a) *hsp*70 and (b) *hsp*90 mRNA of juvenile sea cucumbers in June, July and August. Values are mean ± S.E. (n = 3). Values with different letters are significantly different (*P* < 0.05) among different groups at the same temperature.

Two-way ANOVA results showed that levels of expression of genes encoding heat-shock proteins, *hsps*, of group 4 were significantly higher than those of the other three groups (*hsp*70, *F*_3, 36_ = 23.795, *P* < 0.001; *hsp*90, *F*_3, 36_ = 7.150, *P* = 0.001). However, there was no significant difference in *hsp* expression of individuals collected at different time points (*hsp*70, *F*_2, 36_ = 0.589, *P* = 0.563; *hsp*90, *F*_2, 36_ = 0.430, *P* = 0.857, Fig 8).

**Fig 9:**
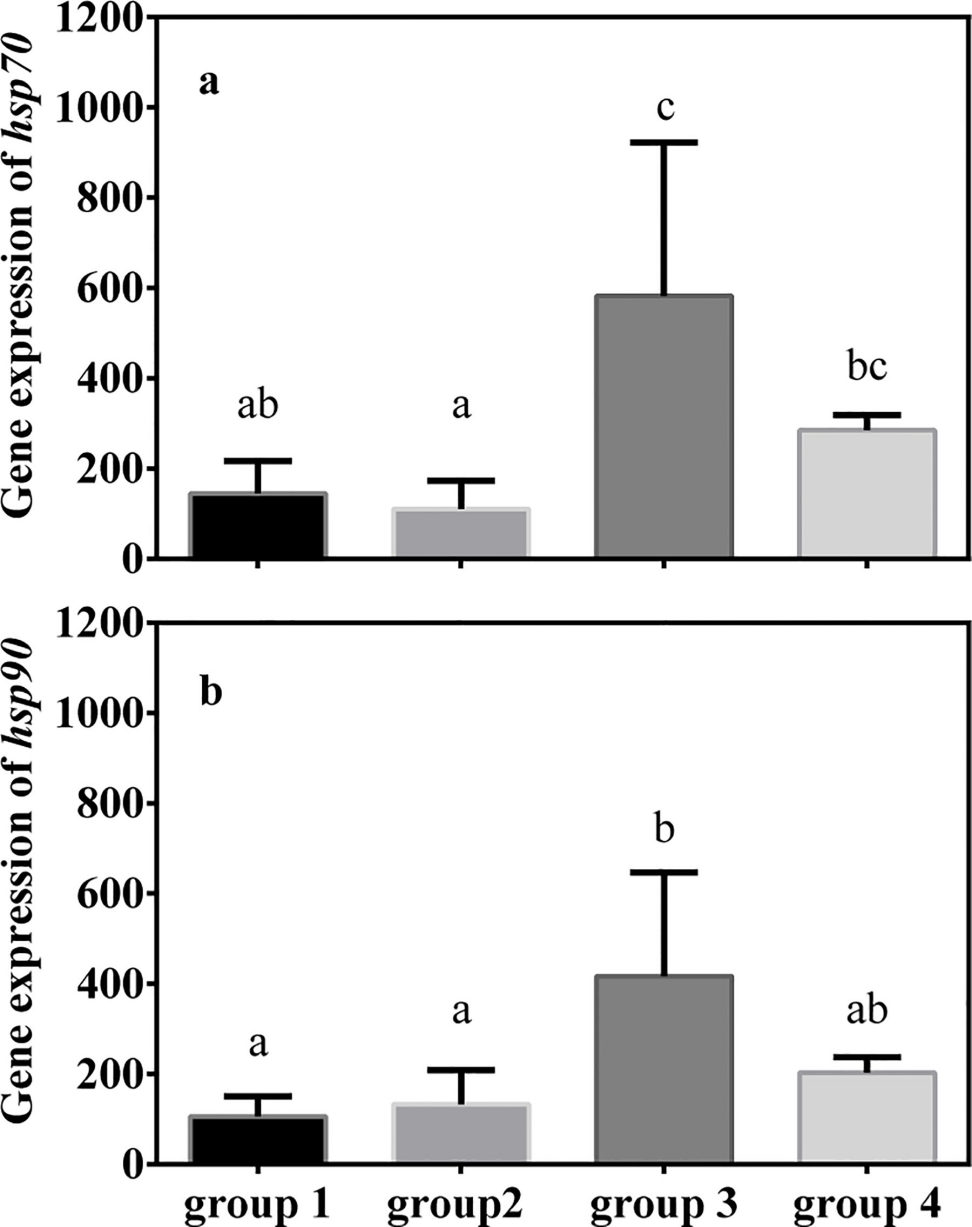
Levels of (a) *hsp*70 and (b) *hsp*90 mRNA of juvenile sea cucumbers after acute thermal stress. Water temperature was increased from 23°C to 29°C within 2h. Values are mean ± S.E. (n = 3). Values with different letters are significantly different (*P* < 0.05).

After acute heat shock at 29°C, genes encoding heat shock proteins were significantly upregulated in all the four groups (Fig 9). The maximum values of *hsp* expression occurred in group 3, and *hsp* levels in group 3 were significantly higher than in group 1 and group 2 (*hsp*70: *F*_3, 11_ = 6.800, *P* = 0.014; *hsp*90: *F*_3, 11_ = 4.108, *P* = 0.049), but there was no significant difference in *hsp* expression between group 3 and group 4.

## Discussion

Two typical sites for sea cucumber farming in China were selected in this study. Qingdao is the natural range of sea cucumber *A*. *japonicus*, and Xiapu, ~1000 km away from the southern limit of natural distribution of this species is the main region for north-to-south sea cucumber culture. Throughout the whole year in 2012, water temperature in Xiapu was 3–7°C higher than that in Qingdao (Fig 1A). Thus, Xiapu can be regarded as an appropriate location to evaluate the impact of future climate change on the marine invertebrate that naturally occurs in northern China.

In the present study, all the adult sea cucumbers used for artificial breeding were collected from the same broodstock in a same pond in Qingdao, and there was no sea cucumber replenishment from 2010 to 2014. Moreover, larvae of the four groups were reared under a common garden condition in field mesocosm in Xiapu. Thus, the difference in thermal tolerance among different groups should be mainly attributed to the divergent thermal history of adult sea cucumbers. Results of Tukey Post hoc analysis showed that mortality of adults in group 2 was significantly lower than that in group 3 and group 4, and there was no statistical difference between group 3 and group 4. But no significant difference in mortality was found in the culture of juveniles among the four groups. In Northern China, ~10% mortality was common for adult sea cucumbers cultured in aquaculture ponds, and this value can reach to 30%-40% due to the high temperature in summer [37]. In the present study, high mortality rates of adults in group 3 and group 4 might result from higher temperature and longer duration of acclimatization. And after the long acclimatization in field mesocosms, surviving adults had the ability to cope with high tempetatures. The acquisition of high thermal resistance could be attributed to the following two reasons. One was the plasticity of thermal tolerance in *A*. *japonicas*, which was indicated several times in previous studies [22,38–40]. The other could be the role of natural selection, and adults that were adaptable to high temperatures survived during the acclimatization. However, due to the lack of genetic diversity data and enough specimens, quantitative genetic analysis was not conducted here, and further study is needed to clarify this causation.

The most important finding in the present study is the existence of a parental effect of long acclimatization on thermal tolerance of juvenile sea cucumbers. Upper lethal thermal resistance can be influenced by a variety of factors including body size, condition factor of the animals, rate of temperature change, and thermal history [33,41]. And among them, thermal history is considered to be the most critical [39,42]. In the present study, LT_50_ values of juvenile sea cucumbers of group 3 (31.146°C) and group 4 (31.842°C) were significantly higher than those of group 1 (30.659°C) and group 2 (30.661°C). Although there was no significant difference between group 1 and group 2, individuals in group 2 acquired a higher induced thermal tolerance than group 1. Interestingly, offspring’s LT_50_ values were linearly related to the time period that elapsed after southward transplantation of parents (Fig 2). These results clearly indicated that acclimatization of parents to different thermal environments can affect the upper thermal limit of their offspring. These results are in line with the beneficial acclimation hypothesis (BAH) [15], which presumes that acclimatization to a particular environment gives an organism a performance advantage in that environment over another organism that has not had the opportunity to acclimate to that particular environment. The causation of the high thermal tolerance of the juveniles might be due to the developmental plasticity, which was closely related to the endocrine hormones and DNA methylation [43, 44]. A detailed genetic structure analysis will be carried out to confirm the genetic homogeneity among the four groups.

Another important finding is that the metabolic response to chronic and acute thermal stress among groups was different. Offspring whose parents experienced high temperature acclimatization had higher resistance of metabolism (OCR) to thermal stress. The oxygen consumption of *A*. *japonicus* is sensitive to high temperature, and when temperature exceeds the critical temperature, OCR decreases with rising temperature [21]. In the present study, OCR in all four groups initially increased in July (26°C), and then decreased in August 2013 (29°C) in the outdoor mesocosms. The *F* values of One-way ANOVA, which was applied to indicate the differences of oxygen consumption rates of juveniles among different seasons within one group, were significantly related to the duration after southward transplantation of parents (Fig 5). These results confirmed that offspring whose parents experienced longer periods of acclimatization at high temperature had less seasonal variations of oxygen consumption rates. Furthermore, comparison analysis between OCR_a_ and OCR_c_ showed that juveniles in group 4 had stable OCRs, indicating that the capability of coping with acute thermal stress was much better than individuals from other groups.

Juveniles whose parents experienced high temperature acclimatization had relatively high level of expression of genes encoding heat shock proteins (*hsp*70 and *hsp*90) from June to July 2012. It is well known that heat shock proteins play important roles for protecting proteins as molecular chaperones [28,37,45]. The constitutive expression of *hsp*s in group 4 indicates that offspring whose parents experienced high temperature acclimatization has a “preparative defense” strategy. Previous studies found that limpets encountering extreme and unpredictable heat stress in the upper intertidal zone had higher levels of constitutive heat shock protein expression than species occurring lower in the intertidal region [31,46]. A similar observation was made in field-acclimatized congeneric turban snails that occur at different vertical positions in rocky intertidal habitats [47]. However, the factors causing high levels of constitutive *hsp* expression in group 4 need to be clarified in future studies.

High-temperature acclimatization can also affect offspring’s heat shock response to acute thermal stress. In the present study, *hsp* expression in all four groups increased dramatically when temperature was increased from 23°C to 29°C within 2 h. Among the four groups, sea cucumbers in group 3 had relatively high *hsp* expression compared to group 1 and group 2, which is in accordance with the relatively high upper thermal limits of group 3. However, *hsp* expression of juveniles in group 4 was not significantly higher than in group 1 and group 2. Considering the high upper thermal limit of group 4, there likely are other physiological mechanisms underlying the ability of this group to survive at high temperatures.

In summary, juvenile sea cucumbers whose parents experienced high temperature acclimatization acquired higher thermal tolerance. With increased duration of parental acclimatization at high temperature, offspring became less sensitive to high temperature, as indicated by higher upper thermal limits, less seasonal variation of oxygen consumption, and relatively stable oxygen consumption between chronic and acute thermal stress. The relatively high level of constitutive expression of genes encoding heat shock proteins is likely to be one mechanisms accounting for increased thermal tolerance. Due to the existence of a parental effect of long acclimatization, the thermal sensitivity of sea cucumbers to elevated temperatures under scenarios of future climate change possibly will be reduced.

## Supporting Information

S1 Fig Configuration of the traditional basket culture system. (a) represents a six-tiered basket traditionally used for sea cucumber culture in south China and (b) represents a tier in the basket culture system.
(TIF)

S1 Table Primer sets designed for qRT-PCR analysis of *hsps* mRNA in sea cucumber *Apostichopus japonicus*. (DOCX)

S2 Table Mortality of adult and juvenile sea cucumbers during acclimatization. (DOCX)

S3 Table Time series data on mortalities of adult sea cucumbers during acclimatization. (DOCX)

S4 Table Survival rate of juvenile sea cucumbers *Apostichopus japonicus* after heat-shocked at 29, 30, 31, 32, 33 and 34°C. (DOCX)

S5 Table Initial and final oxygen concentrations in each group at constant temperatures and after acute heat shock. (DOCX)

## References

[1] Walther GR, Post E, Convey P, Menzel A, Parmesan C. Ecological responses to recent climate change. Nature. 2002; 416: 389 11919621 10.1038/416389a

[2] Parmesan C. Ecological and Evolutionary Responses to Recent Climate Change. Annu Rev Ecol Evol S. 2006; 37: 637–669.

[3] Worm B, Barbier EB, Beaumont N, Duffy JE, Folke C, Halpern B, et al. Impacts of biodiversity loss on ocean ecosystem services. Science. 2006; 314: 787–790. 17082450 10.1126/science.1132294

[4] Loreau M, Naeem S, Inchausti P, Bengtsson J, Grime JP, Hector A, et al. Biodiversity and ecosystem functioning: current knowledge and future challenges. Science. 2001; 294: 804–808. 11679658 10.1126/science.1064088

[5] Miner BG, Sultan SE, Morgan SG, Padilla DK, Relyea RA. Ecological consequences of phenotypic plasticity. Trends Ecol Evol. 2005; 20: 685–692. 16701458 10.1016/j.tree.2005.08.002

[6] Pigliucci M. Phenotypic plasticity: beyond nature and nurture Baltimore: Johns Hopkins University Press; 2001.

[7] Palumbi SR, Barshis DJ, Traylor-Knowles N, Bay RA. Mechanisms of reef coral resistance to future climate change. Science. 2014; 344: 895–898. 10.1126/science.1251336 24762535

[8] Wilson RS, Franklin CE. Testing the beneficial acclimation hypothesis. Trends Ecol Evol. 2002; 17: 66–70.

[9] Angilletta MJ Jr, Bennett AF, Guderley H, Navas CA, Seebacher F, Wilson RS. Coadaptation: A Unifying Principle in Evolutionary Thermal Biology. Physiol Biochem Zool. 2006; 79: 282–294. 16555188 10.1086/499990

[10] Runcie DE, Garfield DA, Babbitt CC, Wygoda JA, Mukherjee S, Wray GA. Genetics of gene expression responses to temperature stress in a sea urchin gene network. Mol ecol. 2012; 21: 4547–4562. 10.1111/j.1365-294X.2012.05717.x 22856327 PMC3866972

[11] Hoffmann AA, Sørensen JG, Loeschcke V. Adaptation of Drosophila to temperature extremes: bringing together quantitative and molecular approaches. J Therm Biol. 2003; 28: 175–216.

[12] Somero GN. The physiology of climate change: how potentials for acclimatization and genetic adaptation will determine ‘winners’ and ‘losers’. J Exp Biol. 2010; 213: 912–920. 10.1242/jeb.037473 20190116

[13] Crill WD, Huey RB, Gilchrist GW. Within-and between-generation effects of temperature on the morphology and physiology of Drosophila melanogaster. Evolution. 1996; 50: 1205–1218. 28565273 10.1111/j.1558-5646.1996.tb02361.x

[14] Angilletta MJ Jr. Thermal adaptation: a theoretical and empirical synthesis New York: Oxford University Press; 2009.

[15] Leroi AM, Bennett AF, Lenski RE. Temperature acclimation and competitive fitness: an experimental test of the beneficial acclimation assumption. P Natl Acad Sci. 1994; 91: 1917–1921. 10.1073/pnas.91.5.1917 PMC43275 8127906

[16] Thor P, Dupont S. Transgenerational effects alleviate severe fecundity loss during ocean acidification in a ubiquitous planktonic copepod. Global change biology. 14 11 2014 Available: http://onlinelibrary.wiley.com/doi/10.1111/gcb.12815/full. Accessed 8 January 2015. 10.1111/gcb.12815 25430823

[17] Ferrer A, Mazzi D, Dorn S. Stay cool, travel far: cold-acclimated oriental fruit moth females have enhanced flight performance but lay fewer eggs. Entomol Exp Appl. 2014; 151: 11–18.

[18] Wan C, Wang D, Wang Q, Wang JX, Wang SH, Deng FH, et al China Fishery Statistical Yearbook: Fishery yearbook in China in 2013. Beijing: China Agriculture Press; 2014.

[19] Dong YW, Dong SL, Tian XL, Wang F, Zhang MZ. Effects of diel temperature fluctuations on growth, oxygen consumption and proximate body composition in the sea cucumber *Apostichopus japonicus* Selenka. Aquaculture. 2006; 255: 514–521.

[20] Dong YW, Dong SL, Ji TT. Effect of different thermal regimes on growth and physiological performance of the sea cucumber *Apostichopus japonicus* Selenka. Aquaculture. 2008; 275: 329–334.

[21] Yang HS, Yuan XT, Zhou Y, Mao YZ, Zhang T, Liu Y. Effects of body size and water temperature on food consumption and growth in the sea cucumber *Apostichopus japonicus* (Selenka) with special reference to aestivation. Aquac Res. 2005; 36: 1085–1092.

[22] Ji TT, Dong YW, Dong SL. Growth and physiological responses in the sea cucumber, *Apostichopus japonicus* Selenka: Aestivation and temperature. Aquaculture. 2008; 283: 180–187.

[23] Dong YW, Dong SL. Induced thermotolerance and expression of heat shock protein 70 in sea cucumber *Apostichopus japonicus* . Fisheries Sci. 2008; 74: 573–578.

[24] Strange K, Vokoun J, Noltie D. Thermal tolerance and growth differences in orangethroat darter (Etheostoma spectabile) from thermally contrasting adjoining streams. Am Midl Nat. 2002; 148: 120–128.

[25] Chatterjee N, Pal A, Manush S, Das T, Mukherjee S. Thermal tolerance and oxygen consumption of *Labeo rohita* and *Cyprinus carpio* early fingerlings acclimated to three different temperatures. J Therm Biol. 2004; 29: 265–270.

[26] Das T, Pal AK, Chakraborty SK, Manush SM, Sahu NP, Mukherjee SC. Thermal tolerance, growth and oxygen consumption of *Labeo rohita* fry (Hamilton, 1822) acclimated to four temperatures. J Therm Biol. 2005; 30: 378–383.

[27] Selvakumar S, Geraldine P. Heat shock protein induction in the freshwater prawn Macrobrachium malcolmsonii: acclimation-influenced variations in the induction temperatures for Hsp70. Comp biochem Phys A. 2005; 140: 209–215. 10.1016/j.cbpb.2005.01.008 15748861

[28] Portner HO. Oxygen- and capacity-limitation of thermal tolerance: a matrix for integrating climate-related stressor effects in marine ecosystems. J Exp Biol. 2010; 213: 881–893. 10.1242/jeb.037523 20190113

[29] Feder ME, Hofmann GE. Heat-shock proteins, molecular chaperones, and the stress response: evolutionary and ecological physiology. Annu Rev Physiol. 1999; 61: 243–282. 10099689 10.1146/annurev.physiol.61.1.243

[30] Tomanek L. The importance of physiological limits in determining biogeographical range shifts due to global climate change: the heat-shock response. Physiol Biochem Zool. 2008; 81: 709–717. 10.1086/590163 18844483

[31] Hofmann GE, Somero GN. Evidence for Protein Damage at Environmental Temperatures-Seasonal-Changes in Levels of Ubiquitin Conjugates and Hsp70 in the Intertidal Mussel Mytilus trossulus. J Exp Biol. 1995; 198: 1509–1518. 9319406 10.1242/jeb.198.7.1509

[32] Dong YW, Miller LP, Sanders JG, Somero GN. Heat-shock protein 70 (Hsp70) expression in four limpets of the genus *Lottia*: interspecific variation in constitutive and inducible synthesis correlates with in situ exposure to heat stress. Biol Bull. 2008; 215: 173–181. 18840778 10.2307/25470698

[33] Lesser MP, Bailey MA, Merselis DG, Morrison JR. Physiological response of the blue mussel *Mytilus edulis* to differences in food and temperature in the Gulf of Maine. Comp Biochem Phys A. 2010; 156: 541–551. 10.1016/j.cbpa.2010.04.012 20427024

[34] Laxminarayana A. Induced spawning and larval rearing of the sea cucumbers, Bohadschia marmorata and Holothuria atra in Mauritius. SPC Beche-de-mer Inform Bull. 2005; 22: 48–51.

[35] Zhang Q, Liu Y. Farming Techniques of Sea Cucumber and Sea Urchin. Qingdao: Qingdao Ocean University Press; 1998.

[36] Wu ZZ. Improved method of NH_4_-N determined by hypobromite oxidation in water. Mar Environ Sci. 2007; 26: 84–87(In Chinese with English abstract).

[37] Wang QL, Dong YW, Dong SL, Wang F. Effects of heat-shock selection during pelagic stages on thermal sensitivity of juvenile sea cucumber, *Apostichopus japonicus* Selenka. Aquacult Int. 2011; 19: 1165–1175.

[38] Mislan KAS, Helmuth B, Wethey DS. Geographical variation in climatic sensitivity of intertidal mussel zonation. Global Ecol Biogeogr. 2014; 23: 744–756.

[39] Meng XL, Ji TT, Dong YW, Wang QL, Dong SL. Thermal resistance in sea cucumbers (*Apostichopus japonicus*) with differing thermal history: The role of Hsp70. Aquaculture. 2009; 294: 314–318.

[40] Wang QL, Dong YW, Qin CX, Yu SS, Dong SL, Wang F. Effects of rearing temperature on growth, metabolism and thermal tolerance of juvenile sea cucumber, *Apostichopus japonicus* Selenka: critical thermal maximum (CTmax) and hsps gene expression. Aquac Res. 2013; 44: 1550–1559.

[41] Baker S, Heidinger R. Upper lethal temperature tolerance of fingerling black crapp ie. J Fish Biol. 1996; 48: 1123–1129.

[42] Hutchison VH. Factors Influencing Thermal Tolerance of Individual Organisms In: Esch GW, McFarlane RW, editors. Thermal ecology II. Springfield: U.S. Nat. Tech. Inf. Serv; 1976 pp. 10–26.

[43] Gilbert SF, Epel D. Ecological developmental biology: integrating epigenetics, medicine, and evolution Massachusetts: Sinauer Associates Sunderland; 2009.

[44] Beldade P, Mateus ARA, Keller RA. Evolution and molecular mechanisms of adaptive developmental plasticity. Mol Ecol. 2011; 20: 1347–1363. 10.1111/j.1365-294X.2011.05016.x 21342300

[45] Tomanek L, Sanford E. Heat-shock protein 70 (Hsp70) as a biochemical stress indicator: An experimental field test in two congeneric intertidal gastropods (Genus: *Tegula*). Bio Bull. 2003; 205: 276–284. 14672982 10.2307/1543291

[46] Dong YW, Williams GA. Variations in cardiac performance and heat shock protein expression to thermal stress in two differently zoned limpets on a tropical rocky shore. Mar Biol. 2011; 158: 1223–1231.

[47] Tomanek L. Variation in the heat shock response and its implication for predicting the effect of global climate change on species' biogeographical distribution ranges and metabolic costs. J Exp Biol. 2010; 213: 971–979. 10.1242/jeb.038034 20190122

