# Supplementary figures and images for: Parental Effect of Long Acclimatization on Thermal Tolerance of Juvenile Sea Cucumber Apostichopus japonicus
Source: PLoS One. 2015 Nov 18;10(11):e0143372. doi: 10.1371/journal.pone.0143372 (PMC4651317; doi:10.1371/journal.pone.0143372)

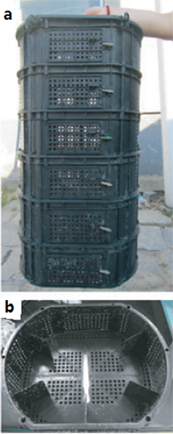

Supplement: S1 Fig — (a) represents a six-tiered basket traditionally used for sea cucumber culture in south China and (b) represents a tier in the basket culture system. (TIF) [file pone.0143372.s001.tif]
