# Supplementary material for: Parental Effect of Long Acclimatization on Thermal Tolerance of Juvenile Sea Cucumber Apostichopus japonicus
Source: PLoS One. 2015 Nov 18;10(11):e0143372. doi: 10.1371/journal.pone.0143372 (PMC4651317; doi:10.1371/journal.pone.0143372)
Supplement: S1 Table — (DOCX) [file pone.0143372.s002.docx]

**S1 Table. Primer sets designed for qRT-PCR analysis of *hsps* mRNA in sea cucumber *Apostichopus japonicus*.**

| Primers | Primer sequences |
| --- | --- |
| Hsp70-F | 5'-AAGAGCACAGGCAAAGAG-3' |
| Hsp70-R | 5'-TGATGATGGGTTGGCACA-3' |
| Hsp90-F | 5'-CTGAACAAAACCAAGCCCATCTG-3' |
| Hsp90-R | 5'-CAAGAGTGCACGGAACTCCAACT-3' |
| 18S rRNA-F | 5'-AGTGAGGATTGACAGATTG-3' |
| 18S rRNA-R | 5'-TTATCGGAATTAACCAGACA-3' |
| β-actin-F | 5'-CATTCAACCCTAAAGCCAACA-3' |
| β-actin-R  Cytb-F  Cytb-R | 5'-TGGCGTGAGGAAGAGCAT-3'  5'-TGAGCCGCAACAGTAATC-3'  5'-AAGGGAAAAGGAAGTGAAAG-3' |

**Note:** Hsp70-F and Hsp70-R were used for amplifying *hsp70*, Hsp90-F and Hsp90-R were used for amplifying *hsp90*, 18S rRNA-F and 18S rRNA-R were used for amplifying 18S rRNA, β-actin-F and β-actin-R were used for amplifying β-actin, and Cytb-F and Cytb-R were used for amplifying Cytb.
