# Supplementary material for: Parental Effect of Long Acclimatization on Thermal Tolerance of Juvenile Sea Cucumber Apostichopus japonicus
Source: PLoS One. 2015 Nov 18;10(11):e0143372. doi: 10.1371/journal.pone.0143372 (PMC4651317; doi:10.1371/journal.pone.0143372)
Supplement: S2 Table — (DOCX) [file pone.0143372.s003.docx]

**S2 Table. Mortality of adult and juvenile sea cucumbers during acclimatization.**

| Group | Replicate | Mortality (%) | |
| --- | --- | --- | --- |
|  |  | Adults | Juveniles |
| 1 | 1 | / | 45.36 |
|  | 2 | / | 52.55 |
|  | 3 | / | 50.31 |
|  | 4 | / | 38.59 |
|  | 5 | / | 49.65 |
| 2 | 1 | 25.00 | 55.22 |
|  | 2 | 16.67 | 48.37 |
|  | 3 | 0.00 | 37.95 |
|  | 4 | 16.67 | 46.44 |
|  | 5 | 16.67 | 59.79 |
| 3 | 1 | 41.67 | 58.66 |
|  | 2 | 33.33 | 33.58 |
|  | 3 | 33.33 | 62.21 |
|  | 4 | 33.33 | 46.69 |
|  | 5 | 16.67 | 54.38 |
| 4 | 1 | 41.67 | 52.74 |
|  | 2 | 50.00 | 49.44 |
|  | 3 | 33.33 | 35.66 |
|  | 4 | 50.00 | 60.21 |
|  | 5 | 33.33 | 39.75 |
