# Supplementary material for: Parental Effect of Long Acclimatization on Thermal Tolerance of Juvenile Sea Cucumber Apostichopus japonicus
Source: PLoS One. 2015 Nov 18;10(11):e0143372. doi: 10.1371/journal.pone.0143372 (PMC4651317; doi:10.1371/journal.pone.0143372)
Supplement: S3 Table — (DOCX) [file pone.0143372.s004.docx]

**S3 Table. Time series data on mortalities of adult sea cucumbers during acclimatization.**

| Group | Replicate | Mortality (%) | | |
| --- | --- | --- | --- | --- |
|  |  | 06/2011-06/2012 | 06/2012-11/2012 | 11/2012-01/2013 |
| 1 | 1 | / | / | / |
|  | 2 | / | / | / |
|  | 3 | / | / | / |
|  | 4 | / | / | / |
|  | 5 | / | / | / |
| 2 | 1 | / | / | 25.00 |
|  | 2 | / | / | 16.67 |
|  | 3 | / | / | 0.00 |
|  | 4 | / | / | 16.67 |
|  | 5 | / | / | 16.67 |
| 3 | 1 | / | 25.00 | 22.22 |
|  | 2 | / | 25.00 | 11.11 |
|  | 3 | / | 25.00 | 11.11 |
|  | 4 | / | 16.67 | 20.00 |
|  | 5 | / | 16.67 | 0.00 |
| 4 | 1 | 25.00 | 11.11 | 12.50 |
|  | 2 | 25.00 | 22.22 | 14.29 |
|  | 3 | 16.67 | 10.00 | 11.11 |
|  | 4 | 33.33 | 12.50 | 14.29 |
|  | 5 | 16.67 | 10.00 | 11.11 |
