# Supplementary material for: Parental Effect of Long Acclimatization on Thermal Tolerance of Juvenile Sea Cucumber Apostichopus japonicus
Source: PLoS One. 2015 Nov 18;10(11):e0143372. doi: 10.1371/journal.pone.0143372 (PMC4651317; doi:10.1371/journal.pone.0143372)
Supplement: S4 Table — (DOCX) [file pone.0143372.s005.docx]

**S4** **Table. Survival rate of juvenile sea cucumbers *Apostichopus japonicus* after heat-shocked at 29, 30, 31, 32, 33 and 34°C.**

| Replicates | Temperature °C | Total number | Survival number | | | |
| --- | --- | --- | --- | --- | --- | --- |
|  |  |  | Group 1 | Group 2 | Group 3 | Group 4 |
| 1 | 29 | 10 | 10 | 10 | 10 | 10 |
|  | 30 | 10 | 9 | 8 | 10 | 10 |
|  | 31 | 10 | 3 | 2 | 6 | 9 |
|  | 32 | 10 | 0 | 0 | 1 | 5 |
|  | 33 | 10 | 0 | 0 | 0 | 2 |
|  | 34 | 10 | 0 | 0 | 0 | 0 |
| 2 | 29 | 10 | 10 | 10 | 10 | 10 |
|  | 30 | 10 | 8 | 9 | 10 | 10 |
|  | 31 | 10 | 3 | 3 | 5 | 8 |
|  | 32 | 10 | 0 | 0 | 0 | 4 |
|  | 33 | 10 | 0 | 0 | 0 | 1 |
|  | 34 | 10 | 0 | 0 | 0 | 0 |
| 3 | 29 | 10 | 10 | 10 | 10 | 10 |
|  | 30 | 10 | 8 | 9 | 10 | 10 |
|  | 31 | 10 | 4 | 4 | 6 | 8 |
|  | 32 | 10 | 0 | 0 | 1 | 3 |
|  | 33 | 10 | 0 | 0 | 0 | 0 |
|  | 34 | 10 | 0 | 0 | 0 | 0 |
