# Supplementary material for: Parental Effect of Long Acclimatization on Thermal Tolerance of Juvenile Sea Cucumber Apostichopus japonicus
Source: PLoS One. 2015 Nov 18;10(11):e0143372. doi: 10.1371/journal.pone.0143372 (PMC4651317; doi:10.1371/journal.pone.0143372)
Supplement: S5 Table — (DOCX) [file pone.0143372.s006.docx]

**S5 Table. Initial and final oxygen concentrations in each group at constant temperatures and after acute heat shock.**

| group | Replicates | Initial oxygen concentration (mg/L) | Final oxygen concentration (mg/L) |
| --- | --- | --- | --- |
|  |  | 23°C 26 °C 29°C 23°C-29°C | 23°C 26 °C 29°C 23°C-29°C |
| 1  2    3 | 1 | 8.03 7.93 7.94 8.00 | 7.10 6.66 7.40 6.76 |
|  | 2 | 7.97 7.95 7.96 8.01 | 7.10 6.75 7.32 6.79 |
|  | 3 | 8.01 7.98 7.98 7.96 | 6.97 6.83 7.41 6.62 |
|  | 1 | 8.02 7.93 7.96 8.01 | 7.18 6.74 7.20 6.79 |
|  | 2 | 7.98 7.94 7.97 8.02 | 7.06 6.82 7.22 6.69 |
|  | 3 | 8.00 7.96 7.99 7.95 | 7.12 6.78 7.23 6.71 |
|  | 1 | 8.02 7.96 7.96 7.96 | 7.27 7.00 7.18 7.00 |
|  | 2 | 7.95 7.98 7.95 8.04 | 7.30 7.07 7.20 6.89 |
|  | 3 | 8.03 7.95 7.99 7.96 | 7.24 6.97 7.28 6.97 |
| 4 | 1 | 8.00 7.97 7.99 8.03 | 7.24 6.98 7.19 7.19 |
|  | 2 | 7.95 7.91 7.93 8.01 | 7.26 7.06 7.20 7.08 |
|  | 3 | 8.04 7.98 7.98 7.94 | 7.33 7.10 7.06 7.00 |
